# Patient characteristics, clinical manifestations, prognosis, and factors associated with gastrointestinal cytomegalovirus infection in immunocompetent patients

**DOI:** 10.1186/s12876-020-1174-y

**Published:** 2020-01-30

**Authors:** Thanaboon Chaemsupaphan, Julajak Limsrivilai, Chenchira Thongdee, Asawin Sudcharoen, Ananya Pongpaibul, Nonthalee Pausawasdi, Phunchai Charatcharoenwitthaya

**Affiliations:** 1 Division of Gastroenterology, Department of Medicine, Faculty of Medicine Siriraj Hospital, Mahidol University, Bangkok, Thailand; 2 Department of Pathology, Faculty of Medicine Siriraj Hospital, Mahidol University, Bangkok, Thailand

**Keywords:** Cytomegalovirus, Gastrointestinal, Immunocompetent, Immunocompromised

## Abstract

**Background:**

Gastrointestinal (GI) cytomegaloviral (CMV) infection is common among patients with immunocompromised status; however, data specific to GI-CMV infection in immunocompetent patients are comparatively limited.

**Methods:**

This retrospective study included patients diagnosed with GI-CMV infection at Siriraj Hospital (Bangkok, Thailand) during 2008–2017. Baseline characteristics, presentations, comorbid conditions, endoscopic findings, treatments, and outcomes were compared between immunocompetent and immunocompromised.

**Results:**

One hundred and seventy-three patients (56 immunocompetent, 117 immunocompromised) were included. Immunocompetent patients were significantly older than immunocompromised patients (73 vs. 48.6 years, *p* < 0.0001). Significantly more immunocompetent patients were in the ICU at the time of diagnosis (21.0% vs. 8.6%, *p* = 0.024). GI bleeding was the leading presentation in immunocompetent, while diarrhea and abdominal pain were more common in immunocompromised. Blood CMV viral load was negative in significantly more immunocompetent than immunocompromised (40.7% vs. 12.9%, *p* = 0.002). Ganciclovir was the main treatment in both groups. Significantly more immunocompetent than immunocompromised did not receive any specific therapy (25.5% vs. 4.4%, *p* ≤ 0.01). Six-month mortality was significantly higher among immunocompetent patients (39.0% vs. 22.0%, *p* = 0.047). Independent predictors of death were old age and inpatient or ICU clinical setting. Treatment with antiviral agents was the only independent protective factor.

**Conclusion:**

GI-CMV infection was frequently observed among immunocompetent elderly patients with comorbidities or severe concomitant illnesses. GI bleeding was the most common presentation. Blood CMV viral load was not diagnostically helpful. Significantly higher mortality was observed in immunocompetent than in immunocompromised patients, but this could be due to more severe concomitant illnesses in the immunocompetent group.

## Introduction

Cytomegalovirus (CMV) is a double-stranded DNA virus in the herpes virus family [1]. CMV is considered an important opportunistic virus among immunocompromised individuals. Patients with human immunodeficiency virus (HIV) infection, immunosuppressive state, long-term steroid or immunomodulator use, and/or organ transplantation are more susceptible to this opportunistic pathogen [2–4]. CMV infection can affect several organs, but gastrointestinal (GI) involvement is one of the most common [5]. GI-CMV manifestations include dysphagia, abdominal pain, diarrhea, and upper or lower GI bleeding [3]. GI-CMV infection in immunocompetent patients was at one time rare; however, there has been an increasing number of case reports and case series of GI-CMV infection in immunocompetent patients, especially among the elderly and critically ill patients [6–28]. Nevertheless, data specific to GI-CMV infection in immunocompetent patients are still quite limited. Only three cohorts comprehensively described risk factors, clinical manifestations, and clinical course [7, 27, 28], and one of those studies included patients taking corticosteroids [27]. This could lead to disease under-recognition, which could result in diagnostic delay and poorer outcomes. Enhanced understanding of GI-CMV infection in immunocompetent patients will improve diagnosis, treatment, and patient outcomes.

Accordingly, the aim of this study was to investigate patient characteristics, clinical manifestations, outcomes and prognoses, and factors associated with GI-CMV infection in immunocompetent patients by comparing those parameters with those of GI-CMV infection patients with immunocompromised status.

## Materials and methods

This retrospective study included patients diagnosed with GI-CMV infection at the Division of Gastroenterology, Department of Medicine, Faculty of Medicine Siriraj Hospital, Mahidol University, Bangkok, Thailand during the January 2008 to December 2017 study period. Siriraj Hospital is Thailand’s largest national tertiary referral center. Cases of GI-CMV infection were identified from the database of the Department of Pathology, Faculty of Medicine Siriraj Hospital, Mahidol University. Only cases with diagnostic confirmation by demonstration of either CMV viral inclusion by hematoxylin and eosin (H&E) staining or by positive immunohistochemistry staining for CMV antigen on pathologic tissue specimens obtained from either endoscopy or surgery were included [29]. Patient demographics, comorbidities, clinical presentations, laboratory investigations (including blood CMV viral load), endoscopic and imaging findings, treatments, and outcomes were collected, recorded, and analyzed. The test for blood CMV viral load was the COBAS® AmpliPrep/COBAS® TaqMan® CMV Test (Roche Molecular System, Inc. USA) which has the range of detection of 150–10,000,000 copies/mL. The protocol for this study was approved by the Siriraj Institutional Review Board (SiRB) on 7 September 2018 (COA no. 566/2561). The requirement to obtain written informed consent from included patients was waived due to the anonymous retrospective nature of this study.

### Definition of immune status

Patients were separated into either the immunocompromised group or the immunocompetent group. Patients with AIDS, with organ transplantation, and/or receiving chemotherapy, systemic corticosteroids, or immunosuppressive agents were defined as immunocompromised in previous reports [7, 30, 31]. All other patients were considered to be immunocompetent.

### Literature review

A PubMed search was performed from its inception to July 2018 to identify/describe the characteristics of GI-CMV disease in immunocompetent patients. Only articles in English were included. The term used for this search were “cytomegalovirus” AND (“gastrointestinal” OR “intestinal” OR “colitis”) AND “immunocompetent”. Eligible articles were reviewed by 2 investigators (JL and CT). Disagreements between investigators were resolved by consensus. In cases where consensus could not be reached, a third investigator (PC) would determine the decision outcome. Only cohorts with at least 10 cases were selected for comparison with our data.

### Statistical analysis

Descriptive statistics were used to summarize patient characteristics. Continuous variables are expressed as median and range or mean ± standard deviation, and categorical variables are presented as number of subjects and percentage. Standard two-group comparison methods were used, including independent *t*-test or Wilcoxon rank-sum test for continuous data, and chi-square test or Fisher’s exact test for categorical data. Mortality rate was compared using log-rank test. Multivariate analysis for factors that significantly predict mortality was performed using Cox regression analysis. A two-tailed *p*-value of < 0.05 was considered significant for all analyses. All analyses were performed using SAS version 9.4 (SAS Institute, Inc., Cary, North Carolina, USA).

## Results

From January 2008 to December 2017, 173 patients with GI-CMV disease were identified, including 56 (32.3%) immunocompetent and 117 (67.7%) immunocompromised patients. Of the 117 immunocompromised patients, 34 (29.1%) had HIV infection with a median CD4 count of 19 cells/mm^3^ (range: 1–187), 24 (20.5%) had organ transplantations, 21 (17.9%) had cancers requiring chemotherapy, 30 (25.6%) and 10 (8.6%) had autoimmune diseases and inflammatory bowel disease (IBD) requiring corticosteroids or immunosuppressive agents, respectively, and 8 had other conditions requiring corticosteroids including 2 with chronic respiratory diseases, 2 with undiagnosed enteritis, 2 with hematologic malignancies but received only palliative corticosteroids not chemotherapy, 1 with sepsis, and the other one with adrenal insufficiency. Ten patients had two underlying condition including 4 with glomerulonephritis undergoing renal transplantation, 3 with hematologic malignancies undergoing bone marrow transplantation, one with HIV and ITP, one with UC and autoimmune hemolytic anemia, and one with malignant thymoma and myasthenia gravis. The medications used among the immunocompromised patients included corticosteroids in 70 (40.5%) patients, immunosuppressive agents in 44 (37.6%) patients, and chemotherapy in 21 (18.0%) patients. Two immunocompetent patients had autoimmune diseases, and one had IBD that required neither corticosteroids nor immunosuppressive therapy.

### Comparison of characteristics between immunocompetent and immunocompromised patients

Comparison of age, gender, underlying diseases, clinical setting status, clinical presentations, laboratory tests, location involvement, endoscopic findings, treatment, and outcomes are shown in Table 1.

**Table 1:** Characteristics of patients with gastrointestinal cytomegaloviral infection

	Immunocompetent (*n* = 56)	Immunocompromised (*n* = 117)	*P*
Age, year (mean ± SD)	73.0 ± 13.9	48.6 ± 16.4	<.0001
Male gender	31 (55.4%)	72 (61.5%)	0.44
Immunocompromised conditions			
HIV infection	0	34 (29.1%) CD4 (median 19, 1–187)	
Transplantation	0	24 (20.5%)	
Cancers receiving chemotherapy	0	21 (17.9%)	
Autoimmune diseases	2 (3.6%) (not receive steroid, immunosuppressant)	30 (25.6%)	
Inflammatory bowel diseases (CD = 3, UC = 8)	1 (1.8%) (not receive steroid, immunosuppressant)	10 (8.6%)	
Other conditions requiring corticosteroids		8 (6.8%)	
^a^10 patients had two underlying conditions			
Medications			
Corticosteroids	0	70 (40.5%)	
Chemotherapy	0	21 (18.0%)	
Immunosuppressive agents	0	44 (37.6%)	
Underlying diseases			
Diabetes mellitus	20 (35.7%)	21 (18.0%)	0.010
Large vessel atherosclerotic diseases	21 (37.5%)	14 (12.0%)	< 0.0001
Chronic kidney disease	13 (23.2%)	28 (23.9%)	0.917
• Stage 3 • Stage 4 • Stage 5	6 (46.2%) 1 (7.6%) 6 (46.2%)	6 (21.4%) 5 (17.9%) 17 (60.7%)	0.244
Cirrhosis	2 (3.6%)	4 (3.4%)	>.99
• Child-Pugh A • Child-Pugh B • Child-Pugh C	0 (0%) 1 (50%) 1 (50%)	1 (25%) 3 (75%) 0 (0%)	0.269
Status conditions at diagnosis			
Clinical Setting			0.024
• Outpatient • Inpatient • Intensive care	23 (41.1%) 21 (37.5%) 12 (21.4%)	69 (59.0%) 38 (32.5%) 10 (8.6%)	
Bacteremia	4 (7.1%)	11 (9.4%)	0.776
Systemic inflammatory response syndrome	29 (52.7%)	53 (45.7%)	0.39
Respiratory failure	20 (35.7%)	25 (21.4%)	0.04
Inotropic drugs	19 (33.9%)	10 (8.6%)	<.0001
Acute renal failure	22 (39.3%)	26 (22.2%)	0.02
Presentation			
Median presenting duration, days (range)	1 (1–60)	10 (1–210)	0.0015
GI bleeding	40 (71.4%)	45 (38.5%)	<.0001
Diarrhea	18 (32.1%)	63 (53.8%)	0.007
Abdominal pain	9 (16.1%)	39 (33.3%)	0.018
Fever	27 (49.1%)	66 (56.4%)	0.369
Severe ileus	4 (7.1%)	3 (2.6%)	0.215
Perforation	1 (1.8%)	3 (2.6%)	>.99
CMV at other organs	0 (0%)	8 (6.8%)	0.055
Investigations			
CMV viral load (*n* = 112)			
• Median (range, IQR)	370 (0–85,599, 4951)	2736 (0–2,988,940, 27,074)	0.010
• CMV VL = 0	11/27 (40.7%)	11/85 (12.9%)	0.002
Location involvement			
Esophagus	4 (7.1%)	14 (12.0%)	0.430
Stomach	10 (17.9%)	32 (27.4%)	0.173
Duodenum	1 (1.8%)	11 (9.4%)	0.106
Jejunum	1 (1.8%)	4 (3.4%)	>.999
Ileum	13 (23.2%)	27 (23.1%)	0.984
Colon (rectum not included)	33 (58.9%)	70 (59.8%)	0.910
Rectum	14 (25.0%)	31 (26.5%)	0.834
Endoscopic findings			
EGD	*N* = 14	*N* = 45	
Ulcer	13 (92.9%)	37 (82.2%)	0.671
Inflammatory mucosa	6 (42.9%)	25 (55.6%)	0.406
Mass	2 (14.3%)	0 (0%)	0.053
Colonoscopy	*N* = 40	*N* = 80	
Ulcer	33 (82.5%)	55 (68.8%)	0.108
Inflammatory mucosa	26 (65.0%)	53 (66.3%)	0.892
Mass	4 (10%)	4 (5.0%)	0.301
Balloon-assisted enteroscopy	N = 1	*N* = 2	
Ulcer	1 (100%)	2 (100%)	
Inflammatory mucosa	1 (100%)	2 (100%)	
Mass	0 (0%)	0 (0%)	
Treatment and outcomes			
Medications			
• Ganciclovir	36/51 (70.6%)	105/114 (92.1%)	0.0003
• Valganclovir	3/51 (5.9%)	15/114 (13.2%)	0.278
• Surgery	6/51 (11.8%)	9/114 (7.9%)	0.424
• None	13/51 (25.5%)	5/114 (4.4%)	<.0001
Median duration of treatment, week (range)	3 (0–6)	3 (0–28)	0.003
Death in 6 months			0.047**
• in 1 month	11/51 (21.6%)	16/114 (14.0%)	
• in 6 months	20/51 (39.2%)	25/114 (21.9%)	
Mucosal healing^c^			
• in 6 weeks	6/9 (66.7%)	7/20 (35%)	0.226
• at or after 6 weeks	8/9 (88.9%)	16/19 (67.9%)	> 0.99

^a^ Ten patients had two underlying condition including 4 with glomerulonephritis undergoing renal transplantation, 3 with hematologic malignancies undergoing bone marrow transplantation, one with HIV and ITP, one with ulcerative colitis and autoimmune hemolytic anemia, and one with malignant thymoma and myasthenia gravis

^b^log-rank test

^c^only the patients who did not have underlying gastrointestinal disease

### Demographic characteristics and underlying diseases

Patients in the immunocompetent group were significantly older than patients in the immunocompromised group (73.0 vs. 48.6 years, respectively; *p* < 0.01). Fifty-five percent of immunocompetent patients and 61.5% of immunocompromised patients were male (*p* = 0.44). Major metabolic comorbidities, such as diabetes and large vessel atherosclerosis, were significantly more prevalent in the immunocompetent group. The HbA1C level was available in 15 of 20 immunocompetent hosts and 17 of 21 immunocompromised hosts. The mean HbA1C level was 7.12% ± 1.51% in the immunocompetent and 7.13% ± 1.45% in the immunocompromised group (*p* = 0.977). The prevalence of chronic kidney disease was 23.2 and 23.9% in the immunocompetent and the immunocompromised group, respectively. The prevalence and stage of chronic kidney disease were not different between the two groups. The prevalence of cirrhosis was 3.6 and 3.4% in the immunocompetent and the immunocompromised group, respectively. The prevalence and Child-Pugh classification of cirrhosis were also not different between the two groups.

### Clinical setting status

The diagnosis of CMV was made during admission in the intensive care unit (ICU) more frequently in the immunocompetent group (12 patients, 21.4%) than in the immunocompromised group (10 patients, 8.6%). Furthermore, the medical conditions at the time of diagnosis appeared to be worse in the immunocompetent group since the rates of respiratory failure (35.7% vs. 21.4%, *p* = 0.04), use of inotropic drugs (33.9% vs. 8.6%, *p* < 0.01), and renal failure (39.3% vs. 22.2%, *p* = 0.02) were significantly higher than in the immunocompromised group.

When defined critical illness by requiring either mechanical ventilator or inotropic agents, 25 immunocompetent and 25 immunocompromised patients were included. Of these patients, GI-CMV was the primary disease in only three immunocompetent (12%) and five immunocompromised patients (20%). The causes of severe illnesses in immunocompetent patients were severe infections or sepsis in 13 patients (52%), malignancy-associated conditions such as undergoing surgery for tumor resection in 3 patients (12%), cardiovascular diseases in 3 patients (12%), and other conditions in 3 patients. For immunocompromised patients, severe infections or sepsis was noted in 8 patients (32%), malignancy-associated conditions, or receiving chemotherapy in 6 patients (24%), HIV-related diseases in 2 patients (8%), and other conditions in 4 patients.

### Clinical presentations

The immunocompetent patients had more acute presenting symptoms than those presented by immunocompetent patients. The median duration of presenting symptoms in the immunocompetent group was 1 day (range: 1–60), which was significantly less than the 10-day (range: 1–210) duration in the immunocompromised group (*p* < 0.01). Moreover, the presenting symptoms were different between groups. Immunocompetent patients were significantly more likely to present with GI bleeding (71.4% vs. 38.5%; *p* < 0.01); whereas, diarrhea and abdominal pain were more common in the immunocompromised group. Eight (6.8%) immunocompromised patients had concomitant extra-gastrointestinal CMV infections, including six patients with retinitis, one patient with radiculomyelitis, and one patient with retinitis and encephalitis. There was no concurrent extra-gastrointestinal CMV infection in the immunocompetent group.

### Blood CMV viral load

Blood CMV viral load was obtained in 112 cases – 27 immunocompetent and 85 immunocompromised patients. The median viral load in immunocompetent patients and immunocompromised patients was 370 and 2736 copies/mL, respectively (*p* = 0.01). Forty percent of immunocompetent patients and 13% of immunocompromised patients had undetectable viral load (*p* < 0.01).

### Location of involvement and endoscopic findings

**Fig. 1:**
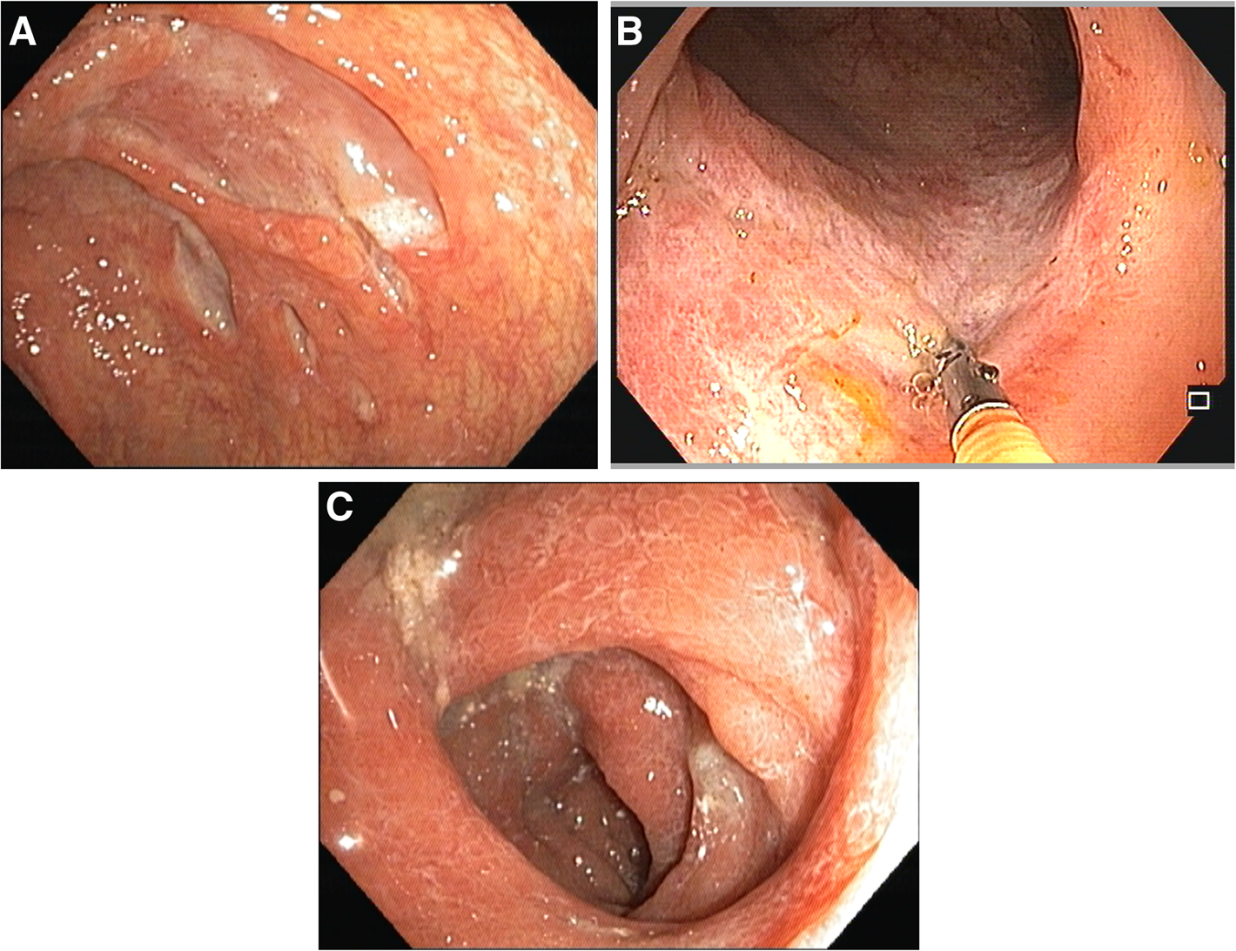
Endoscopic pictures. 1A) Large clean-base ulcers. 1B) Single longitudinal ulcer at sigmoid colon resembling the single stripe sign in ischemic colitis. 1C) Severely inflamed mucosa

In this cohort, CMV infection involved lower GI tract more frequently than upper GI tract in both groups. The immunocompromised group tended to have CMV gastritis more frequently than the immunocompetent group, but the difference between groups was not statistically significant. For endoscopic evaluation, 169 patients had lesions identified during endoscopy, and the tissue biopsies were positive for CMV. Of these, 46 patients had lesions detected by EGD, 107 by colonoscopy, 13 by both EGD and colonoscopy, and three by enteroscopy. The remaining four patients, who did not have endoscopic findings, were diagnosed based on surgical specimens, including one colonic perforation undergoing right hemicolectomy, one colonic obstruction undergoing subtotal colectomy, and two massive ileal bleeding with failed angioembolization undergoing ileal resection. There was no significant difference between groups relative to the finding of endoscopic lesion. Ulcer was the most common type of lesion, with a prevalence of approximately 80–90% in both groups. The ulcers could be either small or large, and they frequently had a clean base (Fig. 1a). Some ulcers had a picture resembling the single stripe sign, which is generally seen in ischemic colitis (Fig. 1b). The intervening mucosa ranged from normal to severely inflamed (Fig. 1c).

### Treatment

Five immunocompetent and 3 immunocompromised patients were referred, which left 51 immunocompetent and 114 immunocompromised patients with available follow-up data. About 70% of immunocompetent patients received antiviral agents. This proportion was significantly less than the proportion in the immunocompromised group, which had a rate of 92% (*p* < 0.01). Ganciclovir was given first to most patients who received antiviral agents, and some patients were switched to valganciclovir. Only 3 patients were started on valganciclovir. More patients in the immunocompetent group (15.7%) required surgery than patients in the immunocompromised group (7.9%) (*p* = 0.13). The proportion of patients who did not receive any specific treatment for CMV infection was significantly higher among immunocompetent patients than among immunocompromised patients (25.5% vs. 4.4%, *p* < 0.01).

### Mortality rate and predictive factors

**Fig. 2:**
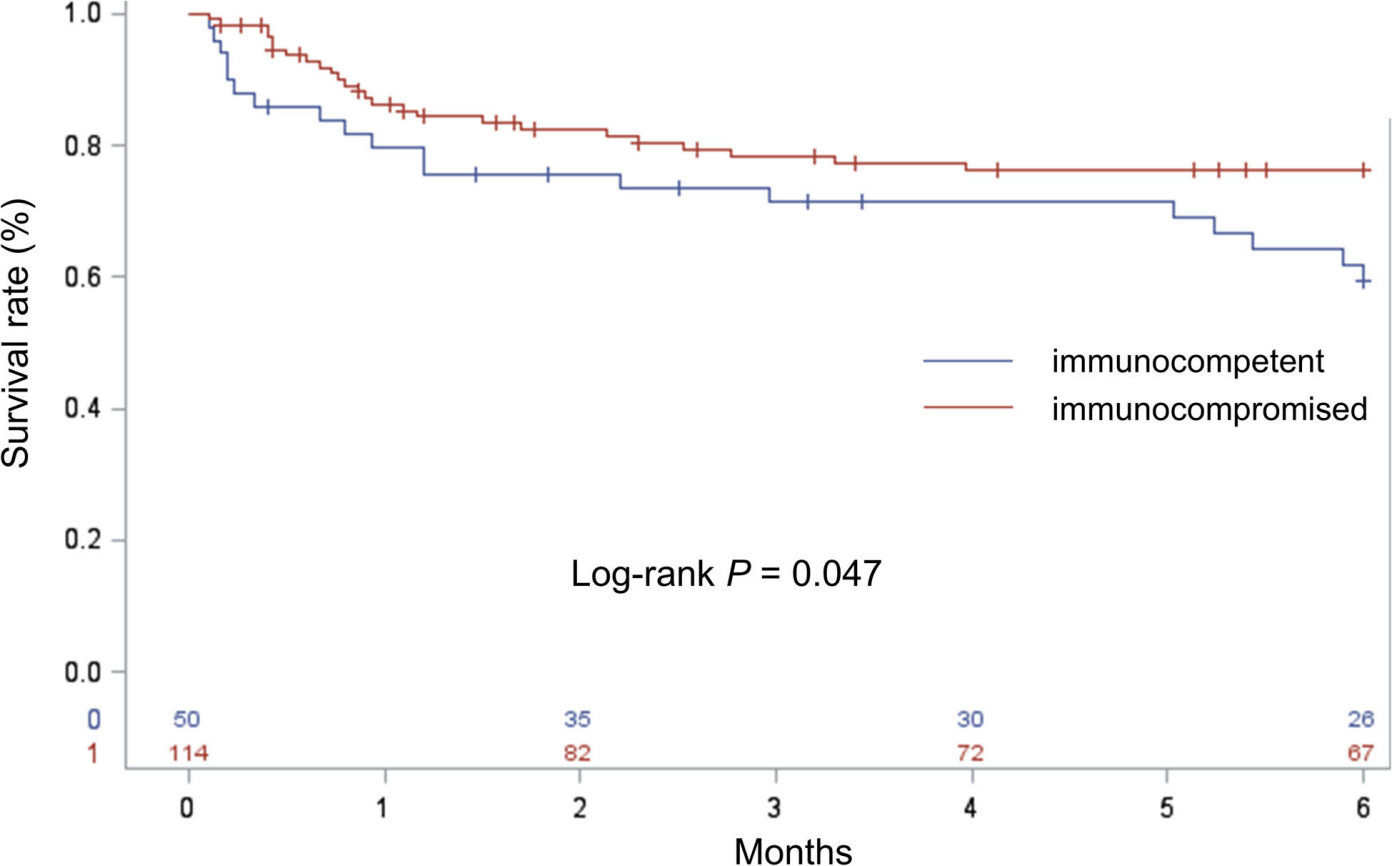
Six-month mortality compared between immunocompetent and immunocompromised gastrointestinal cytomegalovirus infection patients

At the 6-month follow-up time point, 20 (39.2%) immunocompetent and 25 (21.9%) immunocompromised patients had died, and more than half of those patients died within one month. As shown in Fig. 2, the all-cause mortality rate was significantly higher in immunocompetent group (*p* = 0.047).

Multivariate analysis that included age, gender, clinical setting status, presence of systemic inflammatory response syndrome, presence of respiratory failure, presence of acute renal failure, requirement for inotropic drugs, immune status, and receiving antiviral agents, revealed old age, inpatient status, and ICU setting at diagnosis to be independent predictors of 6-month mortality. Treatment with antiviral agents was the only independent protective factor against 6-month mortality, as shown in Table 2.

**Table 2:** Multivariate analysis for predicting 6 month-mortality

Variables	Hazard ratio	95% confident interval	*p*-value
Age	1.021	1.002–1.040	0.03
Status			
• Outpatients	1 (reference)		
• Inpatients	4.31	1.95–9.55	<.01
• ICU	9.14	3.67–22.76	<.01
Antiviral agents	0.24	0.11–0.51	<.01

### Improvement in endoscopic findings

Seventy-seven patients underwent follow-up endoscopy. Of those, 20 patients had underlying gastrointestinal diseases and were not evaluated for mucosal healing. Among the remaining 57 patients who did not have underlying gastrointestinal diseases, the median time of follow-up endoscopy was 6 weeks (range: 1.3–77.3). Among the 29 patients who had endoscopic follow-up before 6 weeks, complete healing was observed in 6 of 9 (67%) immunocompetent patients, and in 7 of 20 (35%) immunocompromised patients (*p* = 0.23). Among the 28 patients who had endoscopic follow-up at or after 6 weeks, complete healing was seen in 8 of 9 (89%) immunocompetent patients, and in 16 of 19 (84%) immunocompromised patients (*p* > 0.99).

### Recurrence of disease

At a median follow-up of 13 months (range: 0.1–111), 7 patients developed recurrent GI-CMV infection. Of those, 5 were immunocompromised patients; 2 had ulcerative colitis that required corticosteroids and immunomodulators; 1 had myasthenia gravis requiring corticosteroids; 1 had myeloid sarcoma that was being treated with allogenic stem cell transplantation; and, 1 patient had AIDS and a CD4 count of 1 cell/mm^3^. One patient in the immunocompetent group developed recurrent GI-CMV infection after receiving corticosteroids for one month for treatment of Crohn’s disease that developed after CMV infection. Another patient in the immunocompetent group suffered from cerebrovascular disease and was bedridden. He developed recurrent GI-CMV infection while stricken with nosocomial sepsis. The median time to recurrence was 4 months (range: 2.3–26.0). The details of these cases are shown in Table 3.

**Table 3:** Recurrent cases

	Underlying disease	Treatment	Follow up endoscopy	Time of recurrence
1	Acute myeloid leukemia s/p allogenic stem cell transplantation with graft-versus-host disease	Ganciclovir 3 weeks	FU at 3 weeks	2.3 months
2	DM, coronary artery disease, cerebrovascular disease, atrial fibrillation with infected bed sore	Ganciclovir 6 weeks	FU at 5 weeks	2.6 months
3	AIDS, CD4 count =1 at the diagnosis	Ganciclovir 6 weeks	No	4 months
4	Myasthenia gravis on corticorsteroid, immunosuppressive agents	Ganciclovir 3 weeks	No	20 months
5	Ulcerative colitis with steroid dependent	Ganciclovir 3 weeks	FU at 11 weeks	26 months
6	Pregnancy with colitis with first diagnosis of CMV and Crohn’s disease. Recurrence occurred after started prednisolone for a month	Ganciclovir 3 weeks	FU at 4 weeks	3.5 months
7	Ulcerative colitis with steroid dependent	Ganciclovir 2 weeks	FU at 11 weeks	23.8 months

### Literature review

The PubMed search using the terms described in the Methods section yielded 181 articles. Our abstract review identified 68 articles that were either case reports or case series of GI-CMV disease in immunocompetent hosts. Of those, 6 case series with at least 10 cases were selected to be reviewed and summarized (Table 4) [7, 14–16, 27, 28].

**Table 4:** Summary of the cohorts of gastrointestinal cytomegaloviral infection in immunocompetent patients (only the cohort with at least 10 patients)

	Ng 1999 (*n* = 10, colitis)	Maiorana, 2003 (n = 11)	Siciliano, 2014 (n = 14, ICU)	Bernard, S 2015 (*n* = 13)	Ko, 2015 (*n* = 51, colitis)	Le 2017 (*n* = 42, colitis)	Current study (*n* = 56)
Age	71	72	64.2	81 (54–88)	65.2 ± 14.0	64.4 ± 19.4	73.0 ± 13.9
Male gender	1 (10%)	9 (88%)	6 (42.8%)	5 (38%)	24 (47.1%)	24 (57.1%)	31 (55.4%)
Underlying disease							
Diabetes mellitus	2 (20%)		5 (35.7%)	3 (23.1%)	15 (29.4%)	15 (35.7%)	20 (35.7%)
Large vessel atherosclerotic diseases	2 (20%)				11 (21.6%) (CVS dis)	8 (19) CAD 8 (19) CVA	21 (37.5%)
Chronic kidney disease	2 (20%)		7 (50%)	2 (15.4%)	16 (31.4%)	6 (14.3%)	13 (23.2%)
Cirrhosis	0		0	0	3 (5.9%)	2 (4.8%)	2 (3.6%)
Cardiomyopathy			9 (64.2%)				
Status conditions at diagnosis							
Status							
• Outpatient						5 (11.9%)	23 (41.1%)
• Inpatient						16 (38.1%)	21 (37.5%)
• Intensive care unit			100%		11 (21.6%)	21 (50%)	12 (21.4%)
Bacteremia						8 (19%)	4 (7.1%)
SIRS						28 (66.7%)	29 (52.7%)
Respiratory failure			71.4%			15 (35.7%)	20 (35.7%)
Inotropic drugs			78.5%			13 (31%)	19 (33.9%)
Acute renal failure						14 (33.3%)	22 (39.3%)
Presentation							
Median presenting duration (days) (range)				8 (1–30)			1 (1–60)
GI bleeding	9 (90%)		10 (71.4%)	6 (46.2%)	30 (58.8%)	22 (52.4%)	40 (71.4%)
Diarrhea	8 (80%)		6 (42.8%)	6 (46.2%)	23 (45.1%)	15 (35.7%)	18 (32.1%)
Abdominal pain	0		1 (7.1%)	0	8 (15.7%)	12 (28.6%)	9 (16.1%)
Fever	6 (60%)		2 (14.3%)	4 (30.8%)	8 (15.7%)	17 (40.5%)	27 (49.1%)
Ileus			1 (7.1%)				4 (7.1%)
Perforation	0		0	0	0	2 (4.8%)	1 (1.8%)
CMV at other organs	0		0	0	0	0	0 (0%)
CMV viremia							
				CMV viral load (n = 4)	Antigenemia (*n* = 30)	pp65 Antigenemia (*n* = 18)	CMV viral load (*n* = 27)
					Median, range 2 (0–11)		Median, range 370 (0–85,599)
				Negative 3/4 (75%)	Negative 13 (43%)	Negative 8 (44.4%)	Negative 11/27 (40.7%)
Treatment and outcomes							
Medications							
• Ganciclovir	3 (30%)		13 (92.8%)	4 (30.8%)	39 (76.5%)	24 (57.1%)	36/51 (70.6%)
• Valganclovir	0			6 (46.2%)		12 (28.6%)	3/51 (5.9%)
• Surgery	1 (10%)		1 (7.2%)	0	5 (9.8%)	6 (14.3%)	8/51 (15.7%)
• None	7 (70%)			7 (53.8%)		12 (28.6%)	13/51 (25.5%)
Duration of treatment (week)				3	2	3	3 (0–6)
Improvement with no treatment	7 (70%)				9 (17.6%)		4 (7.1%)
Death in 6 months			10 (71.4%) (in hospital mortality)	no CMV-related death	7.8% (30 days)	11 (26.2%) (in hospital mortality)	20/51 (39.2%)

## Discussion

This retrospective review of 173 patients diagnosed with GI-CMV during the last ten years at our center revealed that CMV gastrointestinal disease in immunocompetent patients is not rare. In fact, about one-third of GI-CMV infection patients in our hospital did not have obvious immunocompromised status. Furthermore, immunocompetent GI-CMV infection patients were significantly older, had more major metabolic comorbidities, had more severe clinical setting, and commonly presented with gastrointestinal bleeding. CMV viremia was quite uncommon in immunocompetent patients when compared to immunocompromised patients. The mortality rate at 6 months was higher in immunocompetent patients; however, this may be attributable to the significantly older age of immunocompetent patients, and the fact that they had more severe underlying disease.

Like other herpes viruses, CMV causes a primary infection that is followed by a latent infection. Viral DNA has been detected in monocytes, dendritic cells, megakaryocytes, and myeloid progenitor cells in bone marrow [32]. Reactivation causing tissue-invasive diseases usually occur in immunocompromised patients, including those with AIDS, organ transplantation, and those receiving immunosuppressive agents. However, CMV has been reported to cause severe infection in immunocompetent patients, and the GI tract was the most frequent site of infection [33]. Our study showed that the prevalence of GI-CMV infection is not rare since about one-third of GI-CMV patients in our cohort did not have apparent immunocompromised status. This prevalence is similar to that from a previous report by Patra, et al. [1]

To comprehensively identify and describe the disease characteristics, we performed a literature review and obtained details and data from cohorts with at least 10 cases, as summarized in Table 4.

Regarding risk factors, immunocompetent individuals who had GI-CMV infection in our cohort had some conditions that could compromise their immune function. Advanced age is one of the most important risk factors. The immunocompetent patients in our cohort had a mean age of 73 years, which is comparable to the mean age of patients in other cohorts [7, 14–16, 27, 28]. Some underlying diseases may be associated with CMV reactivation. We found that about one-third of our immunocompetent patients had diabetes mellitus (DM) or large vessel atherosclerosis disease, both of which were found to be significantly higher than in the immunocompromised group. A significant proportion of immunocompetent patients in other cohorts were also reported to have these conditions [7, 16, 27, 28, 31]. Chronic kidney disease was found in about 23% of our immunocompetent patients. Although this prevalence was not significantly different between groups in our study, chronic kidney disease was also found to be quite common in other cohorts, with a prevalence ranging from 14 to 50% [7, 14, 16, 27, 28, 31]. Uremia and dialysis may cause dysfunction of B-cell and T-cell lymphocytes, impaired cytokine regulation, and perturbation of mucosal immunity [34]. Interestingly, cirrhosis does not seem to be a risk factor for development of GI-CMV disease. The prevalence of cirrhosis among GI-CMV was reported to range from 0 to 5.9% [7, 16, 27, 28, 31]. Severe critical illness can cause immune paralysis that was reported to be associated with CMV reactivation [35]. Many studies, including ours, found and reported that 20–100% of patients were in an ICU setting [7, 16, 27].

The clinical manifestations of GI-CMV disease in immunocompetent and immunocompromised patients appear to be different. In our study and in other cohorts, the majority of immunocompetent patients presented with gastrointestinal bleeding [7, 16, 27, 28], while diarrhea was the most common presentation in immunocompromised patients [7]. It is known that CMV can infect vascular endothelium resulting in ischemic damage to the mucosa that causes bleeding [36]. In immunocompetent patients, the ischemic process may be potentiated by hypoperfusion state caused by underlying conditions, and this may cause more bleeding. The endoscopic finding of the “single-stripe sign”, which is normally observed in patients with ischemic colitis, was found in some GI-CMV patients and supports this hypothesis (Fig. 1b). Concomitant CMV infection in other organs is extremely rare. Neither our study nor any of the other previous case series found any cases of concomitant CMV infection in other organs [7, 14, 16, 27, 28]. In contrast, concomitant CMV infection in other organs was reported in 7% of immunocompromised patients. This suggests a hypothesis that CMV reactivation in immunocompetent host is a local reactivation rather than a systemic reactivation. This hypothesis could be supported by the results of blood tests for viremia since as high as 40–75% of patients had negative test for viremia, either by Ag detection or by CMV viral load [7, 27, 28].

The role of specific antiviral treatment for immunocompetent patients is still being debated. A 2005 systematic review showed that spontaneous resolution occurred mainly in patients < 55 years of age that had no other comorbidities [31]. Treatment with antiviral agents was reported to have no effect on mortality rate [7]. However, interestingly, and in contrast – treatment with antiviral agents was identified as the only significant protective factor against death in our study. This issue needs to be further investigated in randomized controlled study.

The reported mortality rate ranged from 8 to 71.4% [7, 14, 16, 27, 31]. The differences in mortality among groups is likely explained by differences in patient characteristics. The mortality rate in our cohort at 6 months after diagnosis in immunocompetent patients was 39%, which is significantly higher than the rate in immunocompromised host. However, immune status was not found to be a significant factor in multivariate analysis. The main risk factors for death were old age, inpatient status, and ICU admission at diagnosis.

Mucosal healing was observed in only about half of patients who had endoscopic follow-up before 6 weeks; however, mucosal healing was found in 90% of those who underwent endoscopic follow-up at or later than 6 weeks, with no significant difference observed between the immunocompetent and immunocompromised groups. This suggests that follow-up endoscopy should not be performed too early.

Recurrence was observed in only 5% of patients in this cohort. Six of 7 patients had compromised immune status at the time of disease recurrence. This low rate of disease recurrence may be attributed to recovery of patient immune function after recovery from severe illness, or as a result of treatment with anti-retroviral agents in AIDS patients. These findings suggest that recurrence of CMV infection is most likely to occur in immunocompromised patients, and particularly among those with poor immune function.

The strength of this study is that it is the largest study in GI-CMV in immunocompetent patients. Furthermore, we included all GI-CMV infection patients that were diagnosed at our center during the last 10 years, and this allowed us to determine the relative incidence of GI-CMV compared between immunocompetent and immunocompromised patients. We comprehensively reported clinical manifestations, investigations, clinical progression including relapse rate. We also performed a literature review that permitted us to compare our findings with those from every study that included ten or more patients. Our study also has some limitations. First, the retrospective nature of our study rendered it vulnerable to missing or incomplete data, such as CMV viral load data was not available for all patients. Second, our data was derived from a single center, so our findings may not be generalizable to other centers or healthcare settings. Third and last, patient management was based on the judgment of the treating physicians, so conclusions specific to the effects of treatment could not be drawn.

## Conclusion

GI-CMV infection was frequently observed among immunocompetent elderly patients with comorbidities or severe concomitant illnesses. GI bleeding was the most common presentation. Blood CMV viral load was not diagnostically helpful. Significantly higher mortality was observed in immunocompetent patients than in immunocompromised patients, but this could be due to more severe concomitant illnesses in the immunocompetent group. Anti-viral agents had a positive effect in this study, and could help to decrease the mortality rate in immunocompromised patients, but need further studies.

## Data Availability

The datasets used and/or analysed during the current study are available from the corresponding author on reasonable request.

## Abbreviations

CMV: Cytomegalovirus
GI: Gastrointestinal
IBD: Inflammatory bowel disease
ICU: Intensive care unit
